# Kratom use disorder as a gateway to an opioid use disorder

**DOI:** 10.1192/j.eurpsy.2023.1365

**Published:** 2023-07-19

**Authors:** A. C. Borges, D. Machado

**Affiliations:** SPSM, CHL, Leiria, Portugal

## Abstract

**Introduction:**

Kratom (*Mitragyna speciosa*) is a psychoactive substance native to Thailand and Southeast Asia with stimulant-like effects at lower doses and opioid-like effects at higher doses. Kratom’s chemical composition, specifically mitragynine and 7-hydroxymitragynine, has partial agonist mu-opioid effect and antagonist effects at the kappa- and delta-opioid receptors. It is primarily sought out for stimulant and opioid-like properties and may be used either for its perceived therapeutic effects or as a recreational drug. It is used mainly for symptoms of pain, anxiety, depression, and opioid withdrawal. Regular use of kratom, especially at higher doses, is associated with dependence, tolerance, and withdrawal. Due to its addictive potential, accessibility, and legal status, there have been increasing cases of kratom use disorder. Concerns regarding its potential for abuse and severe adverse effects are rising. The perception that kratom is a milder and less dangerous opioid-like psychoactive substance is supported by the uptake of kratom use as an opiate substitute and is consistent with data on the unimpaired social functioning of regular kratom users.

**Objectives:**

To alert for the importance of kratom consumption as a potential gateway to an opioid use disorder.

**Methods:**

A non-systematic review of the literature was carried out on PubMed. We looked for reviews and case reports published in the last 10 years containing the terms “kratom”, “*Mitragyna speciosa*”, “drug abuse”, “drug addiction”, and “mitragynine”. We also present a clinical case of opioid use disorder.

**Results:**

We report the case of a 38-year-old man that was observed as an outpatient with opioid abuse disorder treated with buprenorphine. He began consuming Kratom about 20 years ago. He learned about Kratom herbal preparations from the plant *Mitragyna speciosa* from internet forums and started to consume oral preparations. Noticing the low side-effects profile, he started to consume Kratom on a daily basis. The main effect of Kratom was to calm down hyperarousal, stop rumination, reduce anxiety, and enhance sociability. The patient did not report major side effects from the consumption but over time tolerance was reached. Knowing that this substance has opioid effects, the patient started to consume opioids like oxycodone in order to obtain Kraton-like effects. Kraton’s use was thus quickly replaced by oxycodone consumption and dependence.

**Conclusions:**

The increasing popularity of kratom has been accompanied by dependence, adverse effects, and withdrawal symptoms following abstinence. Although it could be used for opioid withdrawal, Kraton consumption could be a gateway to opioid consumption and ultimately culminate in dependence.

**Disclosure of Interest:**

None Declared

